# Protective Effects of *Cirsium japonicum* var. *maackii* Flower on Amyloid Beta_25–35_-Treated C6 Glial Cells

**DOI:** 10.3390/life13071453

**Published:** 2023-06-27

**Authors:** Qi Qi Pang, Sanghyun Lee, Eun Ju Cho, Ji-Hyun Kim

**Affiliations:** 1 Department of Food Science and Nutrition, Kimchi Research Institute, Pusan National University, Busan 46241, Republic of Korea; pangqq@pusan.ac.kr; 2 Department of Plant Science and Technology, Chung-Ang University, Anseong 17546, Republic of Korea; slee@cau.ac.kr; 3 Natural Product Institute of Science and Technology, Anseong 17546, Republic of Korea

**Keywords:** *Cirsium japonicum* var. *maackii* flower, C6 glial cells, neuroprotection, amyloid beta, Alzheimer’s disease, phytochemistry

## Abstract

Amyloid beta (Aβ) is a neurotoxic peptide and a key factor causing Alzheimer’s disease. *Cirsium japonicum* var. *maackii* (CJM) has neuroprotective effects, but the protective effects of the flower from CJM (FCJM) on the neural system remain unclear. This study aimed to identify the fraction of FCJM with the highest neuroprotective potential and investigate its protective mechanisms against Aβ_25–35_-induced inflammation in C6 glial cells. The cell viability and generation of reactive oxygen species (ROS) were measured to investigate the positive effect of FCJM on oxidative stress. Treatment with the FCJM extract or fractions increased the cell viability to 60–70% compared with 52% in the Aβ_25–35_-treated control group and decreased ROS production to 84% compared with 100% in the control group. The ethyl acetate fraction of FCJM (EFCJM) was the most effective among all the extracts and fractions. We analyzed the protective mechanisms of EFCJM on Aβ_25–35_-induced inflammation in C6 glial cells using Western blot. EFCJM downregulated amyloidogenic pathway-related proteins, such as Aβ precursor protein, β-secretase, presenilin 1, and presenilin 2. Moreover, EFCJM attenuated the Bax/Bcl-2 ratio, an index of apoptosis, and upregulated the oxidative stress-related protein, heme oxygenase-1. Therefore, this study demonstrated that FCJM improves cell viability and inhibits ROS in Aβ_25–35_-treated C6 glial cells. Furthermore, EFCJM exhibits neuroprotective effects in Aβ_25–35_-induced inflammation in C6 glial cells by modulating oxidative stress and amyloidogenic and apoptosis signaling pathways. FCJM, especially EFCJM, can be a promising agent for neurodegenerative disease prevention.

## 1. Introduction

Alzheimer’s disease (AD) is a neurodegenerative condition characterized by the damage of neurons in the brain, and one of its potential pathogeneses involves the excessive deposition of amyloid beta (Aβ) [1,2]. Senile plaques are formed in the brains of patients with AD and are mainly located in various neuronal tangles, affecting the transmission of nerve signals and causing oxidative damage and apoptosis of nerve cells, thereby resulting in cognitive impairment [3,4]. The main constituent of a senile plaque is Aβ peptide. Its generation is linked to the amyloid precursor protein (APP), which undergoes cleavage via β-secretase (BACE) and γ-secretase, resulting in the production of Aβ [5,6]. The aggregation of Aβ leads to the hyperphosphorylation of the tau protein, formation of neurofibrillary tangles, and overproduction of reactive oxygen species (ROS), ultimately contributing to AD progression [7,8,9].

*Cirsium japonicum* var. *maackii* (CJM) is a perennial herb known for its potential anti-inflammation, anti-hepatitis, aldose-reductase inhibitory effects, and neuroprotective effects [10,11,12,13,14]. In particular, the flower of CJM (FCJM) has shown promising effects in ameliorating skin aging and promoting melanogenesis [15,16], but the protective effects of the FCJM on the neural system remain unclear. A previous study demonstrated that it included the bioactive flavonoids, cirsimarin, cirsimaritin, and hispidulin, with cirsimaritin being the most important [17]. Flavonoids have strong antioxidant activity and can neutralize free radicals, reduce cell oxidative stress damage, and prevent the occurrence of many chronic diseases, such as cardiovascular diseases, cancer, and neurodegenerative diseases. Moreover, flavonoids have anti-inflammatory properties, which can help reduce inflammation and inhibit the release of inflammatory mediators [18,19]. Given these findings, we designed a series of experiments to investigate the potential neuroprotective efficacy of FCJM extract/fractions. This study also targeted to identify the fraction of FCJM with the highest neuroprotective activities and demonstrated its protective mechanisms against Aβ_25–35_-induced neurotoxicity in C6 glial cells.

C6 glial cells, or astrocytes in the brain, have been widely used as cell models in AD research [20,21]. In our previous studies, C6 cells were used in Aβ_25–35_-induced inflammation and oxidative stress experiments [13,22]. While similar studies have been reported on the potential therapeutic effects of CJM on neurotoxicity [14], this present study aimed to specifically focus on the neuroprotective effect of FCJM extract/fractions on Aβ_25–35_-induced inflammation in C6 glial cells via oxidative stress, blood–brain barrier (BBB) function, and the amyloidogenic pathway. We assessed cell viability and ROS production to identify the most effective material among extracts/fractions of FCJM on Aβ_25–35_-induced inflammation in C6 glial cells. Furthermore, we conducted a Western blot to explore its protective effects and mechanisms. These findings may contribute to the understanding of FCJM as a potential therapeutic agent in AD and provide a basis for further research in this area.

## 2. Results

### 2.1. Effects of FCJM Extract and Fractions on Cell Viability

**Figure 1:**
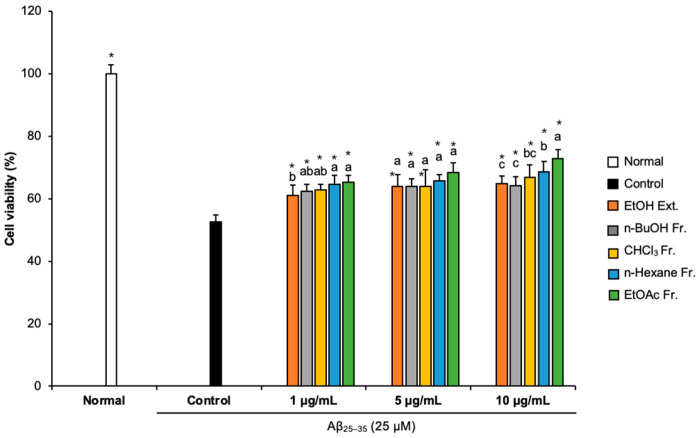
Effect of CJM flower extracts and fractions on cell viability in Aβ_25–35_-treated C6 glial cells. Values are means ± standard deviation. Ext., extract; Fr., fraction. ^a–c^ Means with different letters are significantly different (*p* < 0.05) via Duncan’s multiple range test among extract- and fraction-treated groups. Significant differences between the two groups are observed with Student’s *t*-test (* *p* < 0.001 vs. control). Aβ, Amyloid beta.

We conducted an MTT assay to assess whether FCJM affected cell viability [23]. Compared with the normal group (100%), the cell viability of the Aβ_25–35_-treated control group decreased to 52.59%, indicating that Aβ_25–35_ caused cytotoxicity, which led to cell viability reduction (Figure 1). However, treatment with different concentrations (1, 5, and 10 μg/mL) of FCJM extract and fractions showed an obvious increase in cell viability. In particular, the n-hexane and EtOAc fractions were the most effective in protecting the C6 glial cells from the Aβ_25–35_-induced cytotoxicity among ethanol (EtOH) extract and other fractions. These two fractions of FCJM significantly showed improvement in cell viability within three concentrations (1, 5, and 10 μg/mL, *p* < 0.001, vs. control group). Furthermore, among the extracts and fractions, the EtOAc fraction of FCJM (EFCJM) presented the best improvement in cell viability. These results suggested that FCJM, especially EFCJM, had an ameliorative role in neuronal toxicity in C6 glial cells treated with Aβ_25–35_. Meanwhile, the toxicity of FCJM extract and fractions on cell viability were not detected (Supplementary Materials Figure S1).

### 2.2. Effects of FCJM Extract and Fractions on ROS Production

**Figure 2:**
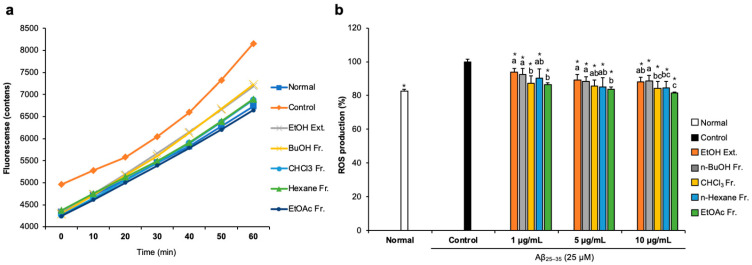
Effect of CJM flower extract and fractions on ROS production in Aβ_25–35_-treated C6 glial cells. (**a**) Time course of the change in the intensity of ROS fluorescence in 60 min. (**b**) The production of ROS in C6 glial cells. Values are means ± standard deviation. Ext., extract; Fr., fraction. ^a–c^ Means with different letters are significantly different (*p* < 0.05) via Duncan’s multiple range test among extract- and fractions-treated groups. Significant differences between the two groups are observed with Student’s *t*-test (* *p* < 0.001 vs. control). Aβ, Amyloid beta; ROS, reactive oxygen species.

To examine whether FCJM possessed antioxidant activity in Aβ_25–35_-treated C6 glial cells, we measured ROS production by detecting 2′,7′-dichlorofluorescein. The fluorescence fluctuation of Aβ_25–35_-treated C6 glial cells was monitored for 60 min (Figure 2a). After treatment with DCF-DA, fluorescence showed a time-dependent tendency in all groups. Notably, Aβ_25–35_-treated C6 glial cells showed higher fluorescence counts, which indicated that the ROS level increased due to Aβ_25–35_ compared to the normal cells. In contrast, treatment with FCJM extracts and fractions showed a time-dependent inhibition in ROS production. Figure 2b presents the fluorescence counts at 60 min. The ROS production was significantly inhibited by EFCJM, which was the most effective material among the other extract and fractions (*p* < 0.001 vs. control). Therefore, these results suggested that FCJM, especially EFCJM, had protective effects on oxidative damage induced by Aβ_25–35_, leading to the inhibition of ROS production.

### 2.3. Regulation of the HO-1 Protein Expression Level by EFCJM in Aβ_25–35_-Treated C6 Glial Cells

**Figure 3:**
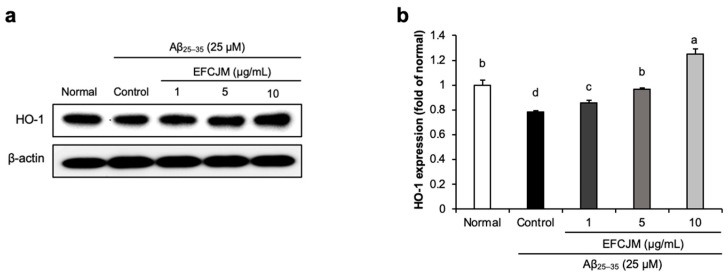
Effects of the ethyl acetate fraction of EFCJM on protein levels of HO-1 in C6 glial cells treated with Aβ_25–35_. (**a**) Bands of HO–1 protein expression. (**b**) HO–1 protein expression level. Values are mean ± standard deviation. ^a–d^ Means with different letters are significantly different (*p* < 0.05), as determined via Duncan’s multiple range test. HO-1, heme oxygenase 1.

To ascertain how FCJM modulates neuroprotective effects, we chose EFCJM, the most effective fraction among the others, and performed Western blotting. We evaluated whether EFCJM regulated HO-1 expression level in Aβ_25–35_- treated C6 glial cells (Figure 3). Our results showed that treatment with Aβ_25–35_ decreased HO-1 expression level compared with that in normal cells. In contrast, treatment with EFCJM dose-dependently increased the HO-1 expression level. These results suggested that EFCJM treatment showed a protective effect on oxidative stress by regulating the HO-1 expression level in Aβ_25–35_-treated C6 glial cells.

### 2.4. Regulation of the Apoptosis-Related Protein Expression Level by EFCJM in Aβ_25–35_-Treated C6 Glial Cells

**Figure 4:**
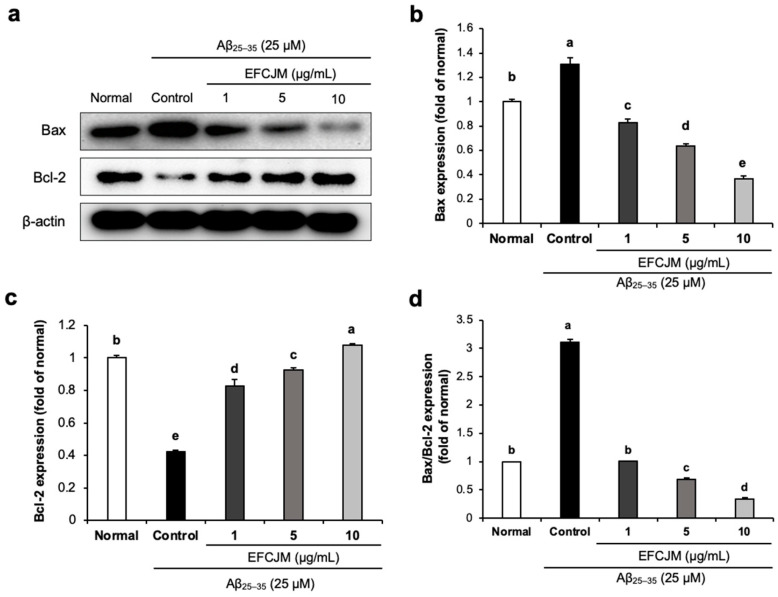
Effects of the ethyl acetate fraction of EFCJM on apoptosis-related protein expression in C6 glial cells treated with Aβ_25–35_. (**a**) Bands of Bax and Bcl-2 proteins expression level. (**b**) Bax and protein expression level. (**c**) Bcl-2 protein expression level. (**d**) Ratio of Bax/Bcl-2 proteins expression level. Values are mean ± standard deviation. ^a–e^ Means with different letters are significantly different (*p* < 0.05), as determined via Duncan’s multiple range test. Bax, B-cell lymphoma 2-associated X protein; Bcl-2, B-cell lymphoma 2.

We also evaluated the effect of EFCJM on improving apoptosis in Aβ_25–35_-treated C6 glial cells. As presented in Figure 4, Aβ_25–35_-treated C6 glial cells showed a significant increase in Bax protein expression and a significant decrease in Bcl-2 protein, which indicated that Aβ_25–35_ activated apoptosis signaling in C6 glial cells. However, treatment with EFCJM decreased the Bax expression level and increased the Bcl-2 expression level. Furthermore, the ratio of Bax/Bcl-2 also showed a significant increase on treatment with Aβ_25–35_ compared with that in normal cells, and treatment with EFCJM decreased the Bax/Bcl-2 ratio, which indicated that EFCJM showed an ameliorating effect on apoptosis. These results suggested that EFCJM ameliorated the Aβ_25–35_-induced apoptosis in C6 glial cells by regulating apoptosis-related proteins.

### 2.5. Regulation of the RAGE and IDE Protein Expression Level by EFCJM in Aβ_25–35_-Treated C6 Glial Cells

**Figure 5:**
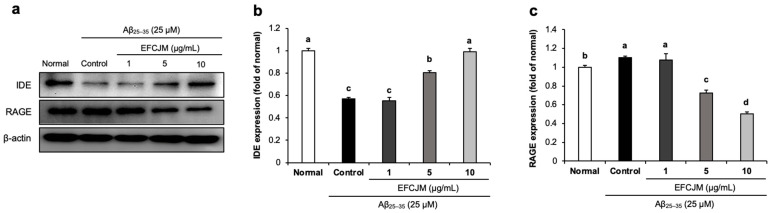
Effects of the ethyl acetate fraction of EFCJM on protein levels of IDE and RAGE in C6 glial cells treated with Aβ_25–35_. (**a**) Bands of IDE and RAGE protein expression. (**b**) IDE protein expression level. (**c**) RAGE protein expression level. Values are mean ± standard deviation. ^a–d^ Means with different letters are significantly different (*p* < 0.05), as determined via Duncan’s multiple range test. RAGE, IDE.

We further investigated whether EFCJM regulated RAGE and IDE protein expression. RAGE acts as a receptor for Aβ and promotes its uptake and transportation, and IDE accelerates Aβ degradation. Figure 5 shows that treatment with Aβ_25–35_ slightly increased RAGE expression and decreased IDE expression. However, after supplementation with EFCJM, a significant decrease was shown in RAGE expression and a significant up-regulation in IDE expression. These results suggested that EFCJM might inhibit Aβ uptake and transportation via the down-regulation of RAGE and promote Aβ degradation via the up-regulation of IDE expression in Aβ_25–35_-treated C6 glial cells.

### 2.6. Regulation of the Amyloidogenic Pathway-Related Protein Expression Level by EFCJM in Aβ_25–35_-Treated C6 Glial Cells

**Figure 6:**
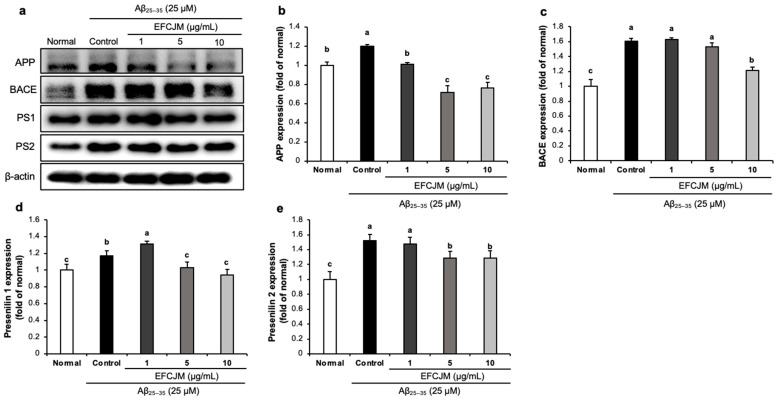
Effects of the ethyl acetate fraction of EFCJM on expression levels of the amyloidogenic pathway proteins in C6 glial cells treated with Aβ_25–35_. (**a**) Bands of amyloidogenic pathway proteins expression. (**b**) APP protein expression level. (**c**) BACE protein expression level. (**d**) Presenilin1 protein expression level. (**e**) Presenilin2 protein expression level. Values are mean ± standard deviation. ^a–c^ Means with different letters are significantly different (*p* < 0.05), as determined via Duncan’s multiple range test. PS1, presenilin 1; PS2, presenilin 2; BACE.

Figure 6 presents the expressions of amyloidogenic pathway-related proteins and the ameliorating effects of EFCJM treatment for each concentration in Aβ_25–35_-treated cells. The expression levels of APP, BACE, PS1, and PS2 were significantly higher in Aβ_25–35_-treated cells than in untreated normal cells. Compared with the EFCJM treated groups, the expressions of APP, BACE, PS1, and PS2 proteins were decreased (1, 5, 10 μg/mL). These results suggested that EFCJM treatment ameliorated Aβ production by modulating amyloidogenic pathway-related proteins.

## 3. Discussion

CJM is presented in the Korean and Chinese pharmacopeias; it is considered a traditional medicine and used as an anti-hemorrhagic, anti-hepatitis, and uretic agent in East Asian regions [24]. The biological activities of CJM have been demonstrated in various chronic disease-related research fields, such as AD, diabetes mellitus, and hypertension [25,26,27]. CJM with medicinal benefits can be consumed as dietary supplements, which can improve health and prevent diseases. In particular, Wagle et al. [26] reported that CJM may be a potential dietary supplement in the treatment of diabetes mellitus and AD. However, the clinical study of CJM on neural systems has not been carried out yet. In 2021, the United States Food and Drug Administration approved aducanumab as the treatment of AD targeting the Aβ plaque. But there was insufficient evidence for drug efficacy and safety [28]. Our study group has investigated and verified the neuroprotective effect of CJM in vitro and in vivo [25,29]. Further, the neuroprotective effects of CJM flowers have not yet been properly studied. In this present study, we focused on the FCJM based on its biological activities, bioactive constituents, and usefulness as a food additive (for example, garnish and tea).

Many studies have shown that the accumulation of Aβ can induce mitochondrial dysfunction and oxidative stress and ultimately lead to cell apoptosis [30,31,32]. Compared with Aβ_1–42_, Aβ_25–35_ causes stronger toxicity and is consequently more suitable for neurotoxicity studies using AD models [33,34]. Meanwhile, as the astrocytes in the brain, C6 glial cells have been used as cell models for AD research after treatment with Aβ_25–35_ [35,36]. Astrocytes are involved in the immune response of the central nervous system. In the early stage of AD, astrocytes are activated in response to the accumulation of Aβ plaques, which in turn release chemokines and cytokines, such as transforming growth factor-β and monocyte chemoattractant protein-1, and respond to pro-inflammatory cytokines, such as interleukin-6 and tumor necrosis factor-α, simultaneously increasing Aβ production and resulting in the inability of the BBB to maintain normal function [37,38,39,40,41].

C6 glial cells are widely used in neuroscience research since they play a crucial role in supporting and regulating neuron functions in the central nervous system and have high stability and repeatability, which makes them widely used in neuroscience research [42]. Moreover, C6 glial cells can release a variety of cytokines and demonstrate a heightened sensitivity to oxidative damage and inflammatory response [43,44]. The stimulation of Aβ to C6 glial cells can cause cytotoxicity and oxidative stress, thereby affecting the survival and apoptosis of C6 glial cells [45,46]. Moreover, C6 glial cells have been widely used to evaluate the effects of various drugs and therapeutic strategies on Aβ-induced cytotoxic and inflammatory responses [47,48]. Therefore, we applied the Aβ-induced C6 glial cell model in our study to evaluate the neuroprotective effect of the CJM flower. Our previous study showed that Aβ_25–35_ treatment downregulated the viability of C6 glial cells [49,50]. Similarly, our data showed that C6 glial cells treated with Aβ_25–35_ showed decreased cell viability. However, after treatment with FCJM extract/fractions, the cell viability was recovered, indicating that FCJM had a protective effect on Aβ_25–35_-induced cell injury. Furthermore, among the EtOH extract and four fractions of FCJM, we found that the EtOAc fraction showed the highest improvement in cell viability. Death of nerve cells is one of the reasons for accelerating the development of AD pathology [51]. Our experimental data suggested that FCJM extract/fractions, especially the EtOAc fraction, had a protective effect on Aβ_25–35_-induced C6 glial cell damage.

Mitochondria is the site of intracellular ROS generation [52]. ROS includes superoxide anion radical (O_2_^−^), hydroxyl radical (·OH), nonradical oxidants hydrogen peroxide (H_2_O_2_), and singlet oxygen (^1^O_2_) [53]. When ROS is overproduced, they continue to act on NADH-link electron transfer to increase O_2_^−^ formation and induce oxidative stress, which causes neurodegenerative disease [54,55]. In the ROS production assay of this present study, we found that treatment with Aβ_25–35_ increased ROS production compared with that in untreated normal C6 glial cells, similar to previous reports [56,57]. Furthermore, our data showed that treatment with FCJM extract/fractions decreased ROS production in Aβ_25–35_-treated C6 glial cells. Among EtOH extracts and four fractions of FCJM, the CHCl_3_ and EtOAc fractions presented excellent ROS inhibition at concentrations of 1 and 5 μg/mL, but the EtOAc fraction showed more effective ROS inhibition at 10 μg/mL. Meanwhile, our previous study showed that FCJM extract/fractions had the ability to scavenge free radicals, such as 2,2-diphenyl-1-picrylhydrazyl, OH, O_2_^−^, and nitric oxide [58]. Additionally, compared with the EtOH extract and other fractions, the EtOAc fraction showed the highest free radical scavenging ability. To conclude, FCJM, especially EFCJM, inhibited ROS production in Aβ_25–35_-treated C6 glial cells because of its free radical scavenging capacity.

The literature studies in relation to CJM include various bioactivities, such as anti-oxidant, anti-inflammatory, anti-fungal, anti-cancer, anti-diabetic, and anti-AD [10,11,26]. Moreover, in our previous study [14], we reported the protective effects of the aerial part of CJM on Aβ_25–35_-treated C6 glial cells regarding cell viability, ROS, inflammation-related proteins (COX-2, IL-1β, and IL-6), and apoptosis-related proteins (Bax and Bcl-2). However, FCJM, the flower part of CJM, has not been studied in biological activities in detail. Hence, in this present study, we investigated the neuroprotective effects of FCJM. The EtOAc fraction prominently showed the strongest neuroprotective activities in cell viability and ROS evaluation. We further investigated the multiple mechanisms for neurodegeneration-related protein expressions.

Therefore, in this present study, considering that EFCJM presented the strongest protective effects against cell damage in the MTT assay, the highest inhibition in ROS overproduction compared with the other extract/fractions of FCJM, as well as the outstanding free radical scavenging capacity, we chose EFCJM to evaluate how it modulates its neuroprotective effects in Aβ_25–35_-treated C6 glial cells. First, to investigate the molecular mechanism of EFCJM in antioxidation, we measured the protein expression of HO-1 after treatment with EFCJM in Aβ_25–35_-treated C6 glial cells. HO-1 is an essential enzyme for heme catabolism; it can split heme and form biliverdin, as well as synthesize carbon monoxide and ferrous iron [59]. Numerous studies have shown that up-regulation of HO-1 protein expression helps prevent cell death and inflammation caused by oxidative stress [60,61,62]. In addition, our previous study presented that treatment with Aβ_25–35_ inhibited HO-1 protein expression in C6 glial cells [22]. In this present study, we found that treatment of C6 glial cells with Aβ_25–35_ downregulated the protein expression of HO-1; however, treatment with EFCJM increased the HO-1 protein expression. These findings suggested that EFCJM could exert its antioxidant effect by regulating the protein expression of HO-1 in Aβ_25–35_-treated C6 glial cells.

Bcl-2 is a typical anti-apoptotic factor in the Bcl family, and Bax is a typical pro-apoptotic factor. Several researchers reported that the ratio of Bax/Bcl-2 plays an important role in mitochondrial function [63,64]. The Bcl-2 family regulates mitochondrial function by controlling the permeability of mitochondrial membranes [65]. Bcl-2 inhibits cytochrome c release at the mitochondrial outer membrane; in contrast, Bax from the cytoplasm is translocated into mitochondria after receiving a death signal and promotes cytochrome c release [66,67]. Many studies reported that Aβ over-deposition in the brain would cause neuronal apoptosis by modulating the expression of the Bax/Bcl-2 ratio and result in cognitive impairment and neurodegenerative disease [68,69]. Moreover, our previous study described that treatment with Aβ_25–35_ increased the expression of Bax and reduced the expression of Bcl-2 in C6 glial cells, which revealed the effects of Aβ_25–35_ on cell apoptosis [70]. In this present study, our data showed that treatment with Aβ_25–35_ increased the ratio of Bax/Bcl-2 expression, and the EFCJM-treated groups showed significantly decreased expression of Bax/Bcl-2. These results suggested that EFCJM improved Aβ_25–35_-induced apoptosis of C6 glial cells by inhibiting the Bax/Bcl-2 expression ratio.

The BBB is a semipermeable chemical barrier that protects the internal stability of the brain from harmful agents in systemic circulation [71]. In the BBB system, the main function of RAGE as a transmembrane protein is to receive and transport Aβ from the extracellular to the intracellular space [72]. A previous study reported that blocking the function of RAGE could be helpful in developing a treatment or prevention technique for AD [73]. Our results showed that treatment with Aβ_25–35_ increased RAGE protein expression compared with that in untreated normal C6 glial cells. However, the EFCJM treatment group showed a dose-dependent decreased RAGE protein expression. Our data suggested that EFCJM might inhibit Aβ transportation by regulating RAGE protein expression.

Aβ_25–35_ is transported to the cell membrane via the RAGE protein, and Aβ_25–35_ induces mitochondrial dysfunction, which increases ROS production and the Bax/Bcl-2 ratio, suggesting that Aβ_25–35_ leads to cell apoptosis. However, treatment with EFCJM showed improvement in Aβ_25–35_-induced mitochondrial dysfunction and cell apoptosis. Therefore, to investigate the potential protective mechanisms of EFCJM, we also examined the clearance function of EFCJM on Aβ_25–35_-treated C6 glial cells. Many studies have shown that the Aβ clearance mechanism is promising in the development of AD therapeutics [74,75,76]. One of the widely known targets is the IDE protein; it is a zinc metalloendopeptidase, has an important physiological role in insulin metabolism, and mainly exists in the mitochondria and peroxidase [77]. Studies reported that Aβ levels were increased in the IDE knockout mice brain, while another study described that increasing IDE expression reduced soluble and insoluble Aβ formation. Another study suggested that IDE played a clearance role by keeping Aβ away from fibrillogens to prevent Aβ deposition in cells [78,79,80]. Moreover, our previous study showed that Aβ_25–35_-treated C6 glial cells presented a decrease in IDE expression [22]. In this present study, we found that treatment with Aβ_25–35_ decreased the expression of IDE in C6 glial cells. However, the treatment with EFCJM increased the IDE protein expression level at concentrations of 5 and 10 μg/mL. Meanwhile, EFCJM treatment at 1 μg/mL showed no changes in IDE protein expression level. We suggest that the concentration of 1 μg/mL EFCJM was too low to cause an up-regulatory effect on IDE expression. Therefore, our present findings suggested that EFCJM might cause a neuroprotective impact on Aβ_25–35_-treated C6 glial cells by modulating IDE protein expression to degrade Aβ.

In the normal stage, signal transduction is developed along the non-amyloidogenic pathway, but in the AD stage, signal transduction is developed along the amyloidogenic pathway [81]. APP is cleaved via BACE, which is the key enzyme, thereby simultaneously producing Aβ and the presenilin proteins (PS1 and PS2) [82]. Accumulated studies reported that APP and BACE were overexpressed in AD models [83,84]. Aβ_25–35_ has been used to build neurotoxicity models for AD research in vitro and in vivo [85,86]. The previous study also used Aβ_25–35_-treated C6 glial cells to build an in vitro model of neuroinflammation for neurotoxicity research [87]. In our present study, we found that APP expression was increased in Aβ_25–35_-treated C6 glial cells. Most of the research on APP was focused on neurotoxicity, and higher expression of APP indicated Aβ_25–35_ induced neurotoxicity in C6 glial cells [88,89]. However, after treatment with EFCJM, the expression of APP was significantly decreased. Moreover, the expression levels of BACE, PS1, and PS2 proteins were also decreased at the concentration of 10 μg/mL compared with those in the control group. Our findings suggested that EFCJM might exhibit a neuroprotective effect by modulating amyloidogenic pathway-related proteins.

## 4. Materials and Methods

### 4.1. Materials

Aβ_25–35_ was purchased from Sigma Aldrich (Saint Louis, MO, USA). Dimethyl sulfoxide (DMSO) was purchased from Daejung (Gyeonggi-do, Republic of Korea). 3-(4,5-Dimethyl-2-thiazolyl)-2,5-diphenyl-2H-tetrazolium bromide (MTT) was purchased from Bio Pure (Kitchener, ON, Canada). 2′,7′-Dichlorofluorescein diacetate (DCF-DA) was purchased from Sigma-Aldrich (Saint Louis, MO, USA). Dulbecco’s modified eagle medium (DMEM), fetal bovine serum (FBS), penicillin-streptomycin, and trypsin-ethylenediaminetetraacetic acid (EDTA) solutions were obtained from Welgene (Daegu, Republic of Korea). Polyvinylidene fluoride (PVDF) membrane was provided by Millipore Co. (Billerica, MA, USA). The radioimmunoprecipitation (RIPA) buffer was provided by Elpis Biotech. (Daejeon, Republic of Korea). Enhanced chemiluminescence (ECL) substrate solution was obtained from Bio-Rad Laboratories (Hercules, CA, USA). We used the following primary antibodies: APP from Sigma Aldrich (Saint Louis, MO, USA); BACE, presenilin 1 (PS1), presenilin 2 (PS2), β-actin, and B-cell lymphoma 2-associated X protein (Bax) from Cell Signaling Technology (Danvers, MA, USA); RAGE and IDE from Santa Cruz (CA, USA). B-cell lymphoma 2 (Bcl-2) and heme oxygenase 1 (HO-1) were from Abcam (Cambridge, UK). The secondary antibodies included anti-rabbit IgG horseradish peroxidase (HRP)-link and anti-mouse IgG HRP-link from Cell Signaling Technology (Danvers, MA, USA).

### 4.2. Sample Preparation

FCJM was obtained from Imsil Herbal Medicine (Imsil, Republic of Korea). It was botanically authenticated by the Korea National Arboretum. FCJM was supplied in a dried form. The dried FCJM (3 kg) was extracted with 15 L EtOH for 3 h at 65–70 °C under reflux, and 470 g of EtOH extract was obtained. The solvent of EtOH extract was removed in vacuo. The extract was partitioned sequentially with n-hexane (17.4 g), chloroform (2.1 g), EtOAc (3.0 g), and n-butanol (7.7 g). FCJM extract or fractions were dissolved in DMSO at a concentration of 0.1 g/mL and diluted with DMEM prior to use. Moreover, in our previous study, the bioactive components of FCJM were analyzed in the reverse-phase HPLC system [15].

### 4.3. Cell Culture

C6 glial cells were obtained from KCLB (Korean Cell Line Bank, Seoul, Republic of Korea; reference number, KCLB No. 10107). These were cultured in 10% (*v*/*v*) FBS and 1% (*v*/*v*) penicillin-streptomycin containing DMEM and incubated under 5% CO_2_/95% air humidity at 37 °C. After culturing the cells for 1–2 days, we discarded the medium, washed the cells with phosphate-buffered saline (PBS, PH 7.4), and then separated the cells with 0.02% ETDA containing trypsin. After centrifugation at 1000 rpm for 3 min, the cells were resuspended in DMEM for subculture and used in our experiments.

### 4.4. Cell Viability Assay

C6 glial cells were seeded at a density of 5 × 10^4^ cells/well in a 96-well plate and cultured in DMEM at 37 °C for 24 h. Meanwhile, Aβ_25–35_ was dissolved in double distilled water at a concentration of 1 mM, incubated at 37 °C for 72 h, and diluted with cell culture medium prior to use. Cells were then incubated with 1, 5, and 10 μg/mL FCJM extract or fractions for 4 h, followed by incubation with 25 μM Aβ_25–35_ for 24 h. The Aβ_25–35_-treated cells were further treated with 5 mg/mL MTT solution for 4 h, and the formazan crystals were dissolved using DMSO solution. After a 30 min interaction with DMSO, the absorbance of each well was measured at 540 nm.

### 4.5. Measurement of ROS Production

C6 glial cells were seeded at a density of 5 × 10^4^ cells/well in a black 96-well plate and incubated for 24 h. The cells were treated with 1, 5, and 10 μg/mL FCJM extract or fractions for 4 h and then incubated with 25 μM Aβ_25–35_ for 24 h. Additionally, cells were incubated with 80 μM DCF-DA for 30 min, and fluorescence (excitation: 480 nm, emission: 535 nm) was continuously measured for 60 min.

### 4.6. Western Blot Analysis

C6 glial cells were treated with EFCJM (1, 5, and 10 μg/mL), and 25 μM Aβ_25–35_ was added for the following experiments. Cells were harvested and lysed in an ice-cold lysis buffer containing RIPA buffer and 1% protease inhibitor cocktail. The mixture was centrifuged, and only the upper layer of protein was used for quantification. The protein (15 ug) was then separated using 8–13% sodium dodecyl sulphate-polyacrylamide gel and transferred to PVDF membranes in a cold transfer buffer for 2 h at 90 V. The membranes with the transferred proteins were incubated at 4 °C overnight with primary antibodies (APP, 1:1000, catalog number A8717; BACE, 1:1000, catalog number 5606; PS1, 1:1000, catalog number 5643; PS2, 1:1000, catalog number 9979; RAGE, 1:500, catalog number sc-365154; IDE, 1:500, catalog number sc-393887; Bax, 1:500, catalog number 2772; Bcl-2, 1:500, catalog number ab32124; HO-1, 1:1000, catalog number ab13243). The following day, the membranes were incubated with secondary antibodies (anti-Rabbit IgG, 1:1000, catalog number 7074; APP, BACE, PS1, PS2, Bax, Bcl-2, HO-1 anti-mouse IgG, 1:1000, catalog number 7076; IDE; and RAGE) for 1 h at room temperature and then treated with an enhanced chemiluminescence solution and imaged using a chemiluminescence imaging system (Davinch-ChemiTM, Davinchi-K, Seoul, Republic of Korea). The original western band images were shown in Supplementary Materials from Figure S2–S5.

### 4.7. Statistical Analysis

All data are presented as means ± standard deviations. Statistical significance was checked using one-way analysis of variance, followed by Duncan’s multiple tests (*p* < 0.05). Significant differences between the two groups were observed using Student’s *t*-test (* *p* < 0.001 vs. control).

## 5. Conclusions

In summary, our results suggested that extract or fractions of FCJM inhibited Aβ_25–35_-induced ROS production and increased cell viability. The neuroprotective activities, which reside mainly in EFCJM, thus justify its application as a promising agent for AD treatment. Meanwhile, there is a need to compare EFCJM with the major pure bioactive compounds in the EFCJM for further study.

## Data Availability

The data presented in this article are available.

## Supplementary Materials

The following supporting information can be downloaded at: https://www.mdpi.com/article/10.3390/life13071453/s1, Figure S1: Effect of CJM flower extract and fractions on C6 glial cells; Figure S2: Effects of EFCJM on protein levels of HO-1 in C6 glial cells treated with Aβ25–35; Figure S3: Effects of EFCJM on apoptosis-related protein expression in C6 glial cells treated with Aβ25–35; Figure S4: Effects of EFCJM on protein levels of IDE and RAGE in C6 glial cells treated with Aβ25–35; Figure S5: Effects of EFCJM on expression levels of the amyloidogenic pathway proteins in C6 glial cells treated with Aβ25–35.
