# Supplementary figures and images for: Protective Effects of Cirsium japonicum var. maackii Flower on Amyloid Beta25–35-Treated C6 Glial Cells
Source: Life (Basel). 2023 Jun 27;13(7):1453. doi: 10.3390/life13071453 (PMC10381248; doi:10.3390/life13071453)

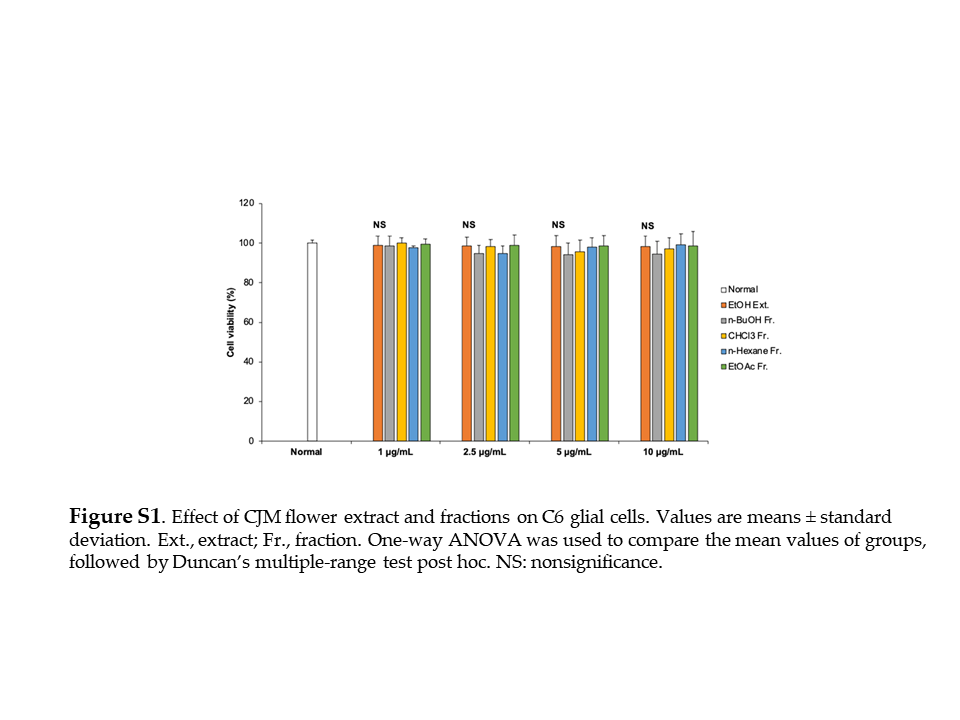

Supplement: Supplementary file 1 [file life-13-01453-s001.zip › life-2342516-supplementary/Figure S1.PNG]

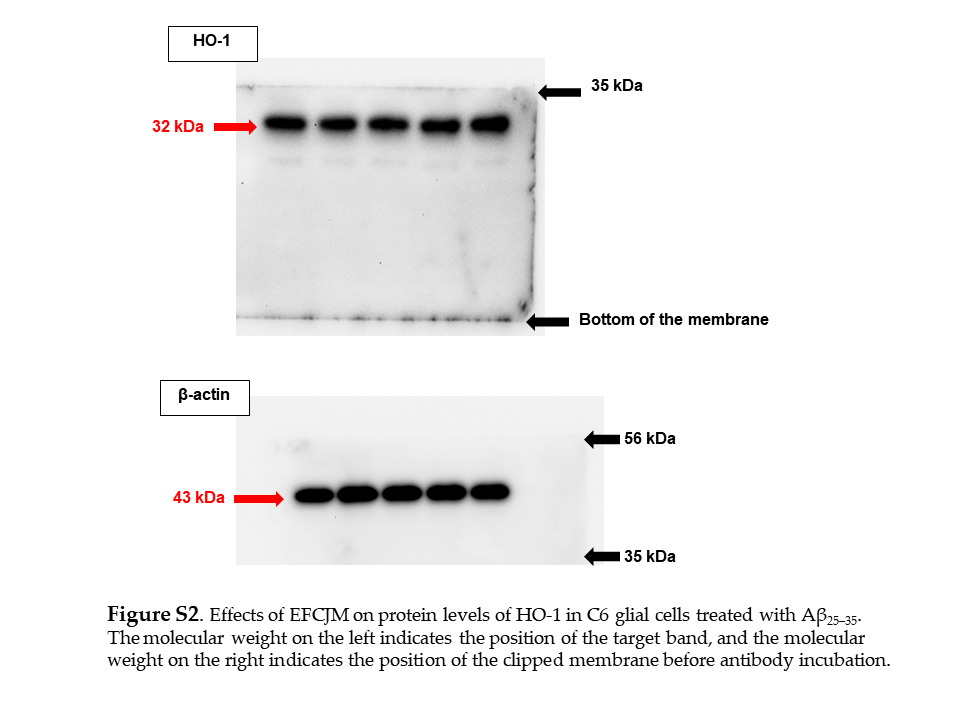

Supplement: Supplementary file 1 [file life-13-01453-s001.zip › life-2342516-supplementary/Figure S2.PNG]

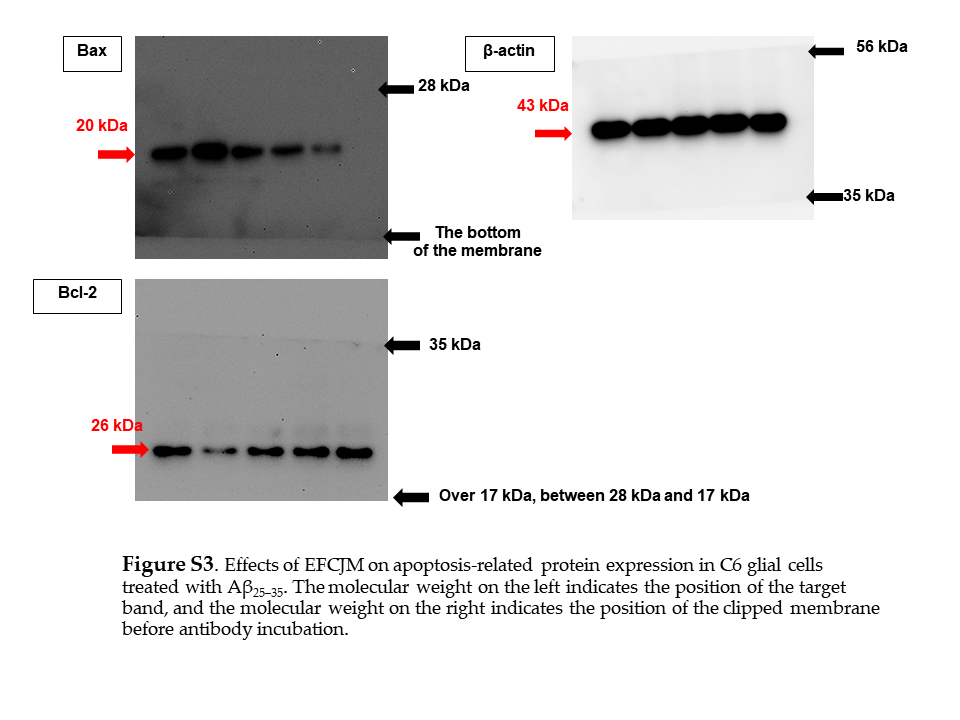

Supplement: Supplementary file 1 [file life-13-01453-s001.zip › life-2342516-supplementary/Figure S3.PNG]

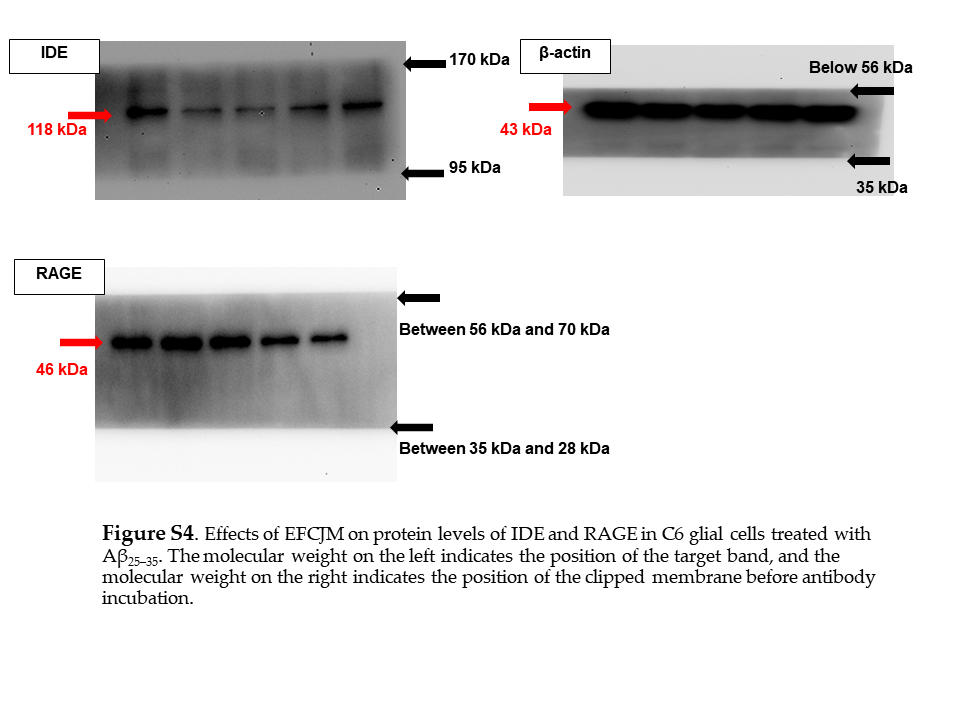

Supplement: Supplementary file 1 [file life-13-01453-s001.zip › life-2342516-supplementary/Figure S4.PNG]

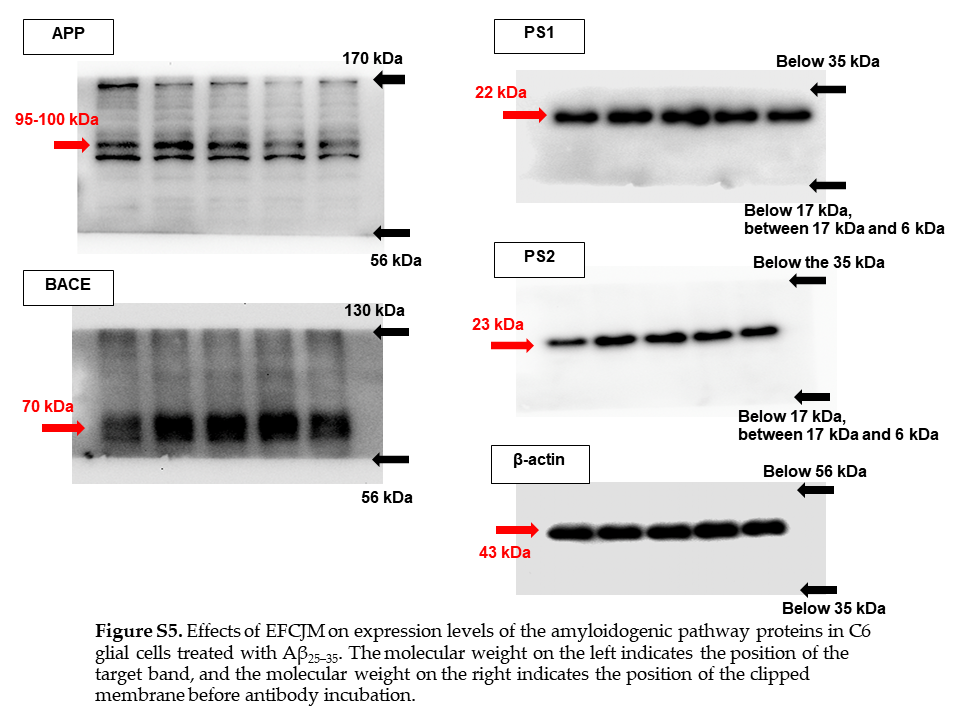

Supplement: Supplementary file 1 [file life-13-01453-s001.zip › life-2342516-supplementary/Figure S5.PNG]
